# Postural control in humans: a study using transcutaneous spinal cord stimulation

**DOI:** 10.1113/EP093385

**Published:** 2025-11-22

**Authors:** Natalia Shamantseva, Ivan Sakun, Tatiana Klishkovskaia, Andrey Aksenov, Vsevolod Lyakhovetskii, Tatiana Moshonkina

**Affiliations:** ^1^ Laboratory of movement physiology Pavlov Institute of Physiology Russian Academy of Sciences St. Petersburg Russia; ^2^ Laboratory of motion capture and simulation systems Saint Petersburg Electrotechnical University ‘LETI’ Saint Petersburg Russia; ^3^ Department of leather products design Saint Petersburg State University of Industrial Technologies and Design Saint Petersburg Russia

**Keywords:** body kinematics, cognitive style, electrical stimulation, external breathing, muscle coactivation, postural control, spinal cord

## Abstract

The aim of the study was to investigate the spinal mechanisms involved in regulating postural balance in humans. Participants stood in a normal stance, with their spinal postural networks either non‐invasively activated or not stimulated by electrical stimulation. Postural sway, muscle activity, joint kinematics and respiratory movements were monitored. Half of the participants depended on external sensory cues for controlling balance (field dependent), whereas the other half did not (field independent). Stimulation was performed at the T11–T12 or L1–L2 vertebral level using intensities below the motor threshold. When participants stood without stimulation, differences in body segment movement in the frontal plane were observed between subgroups, in addition to similarities in segment movement in the sagittal plane. Field‐dependent participants demonstrated greater mediolateral sway and pelvic motion and relied more on hip involvement in frontal centre of pressure dynamics than field‐independent participants. Stimulation at T11–T12 induced changes in muscle activity and body segment coordination in both groups. It led to a reduction in mediolateral sway and enhanced postural stability, but only in field‐dependent individuals. Stimulation at L1–L2 altered muscle activity and joint kinematics in both subgroups without having an effect on postural stability. Stimulation did not affect respiratory movements or posture–respiratory coupling. In the human spinal cord, the interneuronal networks involved in postural regulation are located at the T11–T12 level and operate under supraspinal control. Interneuronal networks at the L1–L2 level also modulate muscle activity and body segment coordination; however, their specific role in regulating upright posture remains unclear.

## 1 INTRODUCTION

Transcutaneous spinal cord stimulation (tSCS) is a non‐invasive neuromodulation technique that modulates spinal circuits by recruiting low‐threshold, large‐diameter afferents, thereby activating motor networks and motoneurons (Gerasimenko et al., 2015; Gorodnichev et al., 2012). At higher intensities, smaller‐diameter afferents are also recruited, engaging additional intraspinal connections, whereas stimulation at or below the motor threshold modulates corticospinal excitability (Benavides et al., 2020; Massey et al., 2024; Shamantseva & Moshonkina, 2025). tSCS is a convenient technology for investigating spinal locomotor networks in healthy individuals (Bikchentaeva et al., 2024; Carrera et al., 2022; Omofuma et al., 2024).

Thoracolumbar tSCS, when delivered at motor threshold intensities, has been shown to enhance postural stability and trunk control in individuals with spinal cord injury (Sayenko et al., 2019). These effects are frequency dependent and involve both modulation of spinal excitability and muscle activation. Stimulation at 5 Hz primarily targets motoneurons via Ia afferents, whereas 15–25 Hz recruits broader spinal interneuronal circuits, supporting better multisegmental coordination and more effective self‐assisted standing (Sayenko et al., 2019).

In healthy individuals, thoracolumbar tSCS has been shown to modulate postural control in a segment‐specific manner. When applied at the L1–L2 vertebral level, tSCS increased postural sway by ≤30% (Shamantseva et al., 2023). In contrast, stimulation at the T11–T12 level led to postural stabilization (Shamantseva et al., 2024). Furthermore, application of tSCS at T11–T12 during destabilizing auditory input has been shown to reduce lateral sway, suggesting an influence on supraspinal mechanisms via modulation of spinal locomotor networks (Shamantseva et al., 2024).

Individual variability in postural responses to tSCS has been explained, in part, by cognitive style, specifically, the distinction between field‐dependent (FD) and field‐independent (FI) individuals (Witkin & Goodenough, 1981). FD individuals tend to rely more on external sensory cues (exteroceptive input) for spatial orientation, whereas FI individuals rely more on internal cues (interoceptive input). Prior studies in postural control have shown that, in the absence of visual input, FD individuals often use an ‘*en bloc*’ stabilization strategy, coupling head–shoulder–hip segments, whereas FI individuals maintain more independent segmental motion (Isableu et al., 2003, 2010). FD participants not only showed greater sway and instability in challenging stance conditions, but also experienced more frequent falls than FI participants, underscoring the functional importance of cognitive style for maintaining balance (Isableu et al., 2010). These differences reflect distinct modes of sensorimotor integration and might influence how spinal and supraspinal circuits interact when external sensory inputs are limited. Recent work indicates that tSCS modulates postural sway in FD but not FI individuals (Shamantseva et al., 2023, 2024). It was speculated that in FD participants, tSCS at the T11 vertebral level improved vertical stability, probably owing to increased hip joint stiffness (improved shoulder–hip coupling), and tSCS at the L1 level destabilized vertical stability by increasing the stiffness of ankle joints in a similar way to that of a mechanical restriction of these joints. In the FI participants, changes in the activity of muscle groups during stimulation were likely to be compensated by independent movements in the joints (Isableu et al., 2003, 2010).

In addition to posture, tSCS at thoracic or lumbar levels has been reported to influence respiration (Minyaeva et al., 2017, 2019). The stimulation can activate spinal breathing networks and/or motor pools innervating respiratory muscles (Shandybina et al., 2022). In a study with healthy participants, it was evident that tSCS at T11–T12 vertebrae inducing stepping movements increased the breathing frequency and reduced tidal volume (Minyaeva et al., 2017). Follow‐up studies indicated that the decrease in tidal volume during tSCS at the T11–T12 level was attributable to the tonic contraction of the abdominal muscles. This might be caused by direct stimulation of the abdominal muscles by bipolar electrical pulses or activation of expiratory motoneurons via descending pathways (Minyaeva et al., 2019).

Given that tSCS at the T11–T12 level can influence external respiration, it is conceivable that its effects on postural control in upright standing are mediated not only through activation of postural muscle networks but also via changes in respiratory kinematics. In healthy adults, small angular shifts of the trunk and hips actively compensate for respiratory‐induced postural perturbances, resulting in minimal posture–respiratory synchronization (Gurfinkel et al., 1971; Hodges et al., 2002; Manor et al., 2012). It has been shown that postural perturbations and physical tasks can indirectly affect respiratory muscles, thus challenging ventilation (Gandevia et al., 2002). However, even in the absence of postural disturbance, the CNS coordinates respiratory and postural demands through shared muscle control, particularly involving the diaphragm, which contributes to both ventilation and trunk stability (Gandevia et al., 2002). To understand the spinal mechanisms that regulate upright posture, it is necessary to monitor both the centre of pressure (CoP) projection movements and respiratory movements.

The specific mechanisms through which spinal neural networks regulate human posture remain largely unexplored. Previous studies have shown that activating the spinal cord at one vertebrae level stabilizes posture (Т11–Т12), whereas activating it at another level destabilizes it (L1–L2). In this study, we simultaneously monitored postural sway, muscle activity, body segment motion and breathing movements during tSCS in the standing position. To isolate the direct effects of tSCS on respiratory movements independent of postural modulation, we also assessed breathing dynamics in the seated position. We hypothesized that: (1) tSCS at the T11–T12 level would reduce postural sway (i.e., stabilize posture) in FD individuals by increasing hip joint stiffness; (2) tSCS at the L1–L2 level would increase postural sway (i.e., destabilize posture) by increasing ankle joint stiffness; and (3) stimulation of these spinal loci would not alter respiratory movements, meaning that the recorded changes in posture would be related solely to modulation of spinal locomotor networks.

By integrating postural, neuromuscular and respiratory measures, the results of this study contribute to the understanding of spinal mechanisms involved in postural balance regulation in humans.

## 2 MATERIALS AND METHODS

The procedures and studies were conducted in October and November of 2024 in compliance with the principles of the *Declaration of Helsinki* and received approval from the Ethics Committee of the Pavlov Institute of Physiology of the Russian Academy of Sciences (Minutes #24‐01, dated 29 March 2024). All the subjects provided written informed consent.

### 2.1 Participants

The inclusion criteria were a body mass index of 18.5–24.9 kg/m^2^, an age of 18–35 years, a Tiffeneau–Pinelli index of ≥80%, and a dominant right leg. The exclusion criteria were any respiratory or musculoskeletal disorder, any symptoms of any kind of disease, medical/surgical procedure or trauma within 4 weeks of the initiation of the study, scoliosis, hernias, vestibular disorders, pregnancy, and a history of epilepsy.

### 2.2 Procedures and tasks

Prior to the study protocol, the Tiffeneau–Pinelli index was assessed as a screening tool to assess the normality of lung function. This index is calculated as the ratio obtained by dividing the forced expiratory volume in 1 s by the forced vital capacity of the lungs and is used for respiratory monitoring (Bhatt et al., 2019). A clinical spirograph Diamant KM‐AP‐01 (Diamant LLC, Moscow, Russia) was used.

The cognitive style of the participants was determined using the Group Embedded Figures Test modified by Gottschaldt (1929). This pencil‐and‐paper test is the most frequently used assessment for field dependence/independence (Hayes & Allinson, 1994). In the test, participants were asked to identify one of five reference figures hidden within 30 complex, masked figures. These figures were presented one at a time, and the total completion time was recorded. Higher accuracy and shorter completion times indicated greater field independence. Based on their Gottschaldt coefficient values, participants were classified as FI (coefficient > 2.5) or FD (coefficient < 2.5). The threshold value is derived from the Group Embedded Figures Test modified by Gottschaldt (1929) and has been used empirically in previous studies to distinguish between FD and FI individuals (Andreeva et al., 2018; Shamantseva et al., 2024; Shoshina & Shelepin, 2014).

The study consisted of six recordings: three in a standing position with and without tSCS, and three in a sitting position. Half of the participants started the investigation with the sitting position, and the other half with the standing position. There were three experimental conditions in each position: control without stimulation, tSCS at the T11–T12 vertebrae level (T11 tSCS) and tSCS at L1–L2 vertebrae level (L1 tSCS). Half of the participants started with T11 tSCS, half with L1 tSCS (Table 1). During all the recordings, participants’ eyes were covered with a mask and their ears were plugged with earplugs. Each recording lasted 100 s.

**TABLE 1:** Experimental design.

Position	Experimental conditions	Notes on counterbalancing
Standing	(1) Control (no tSCS) (2) T11–T12 (L1–L2) tSCS (3) L1–L2 (T11–T12) tSCS	Order of positions and T11/L1 stimulation was counterbalanced across participants
Sitting	(1) Control (no tSCS) (2) T11–T12 (L1–L2) tSCS (3) L1–L2 (T11–T12) tSCS	

Abbreviation: tSCS, transcutaneous spinal cord stimulation.

**FIGURE 1:**
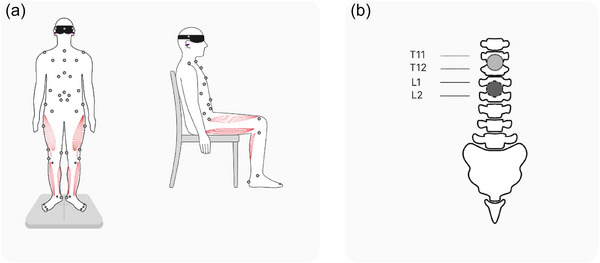
(a) Experimental set‐up for standing and sitting positions. Miniature circles represent motion‐capture markers, and pink shapes represent muscles recorded by electromyography. (b) The position of two cathodes for transcutaneous spinal cord stimulation relative to vertebrae.

Participants stood on a force plate in a standard position (heels together, toes apart, and arms down by their sides; Figure 1a). Foot placement was controlled by the investigator and guided by a marked scheme on the platform to ensure that the same stance was reproduced in each recording.

During the seated recordings, the participants’ hips, knees and ankles were bent at 90°, and the chair supported their backs vertically. The hands were kept hanging by the sides to avoid covering the markers on the chest (Figure 1a).

EMG, stabilometry and motion capture was recorded simultaneously while standing. EMG and motion capture were recorded simultaneously while sitting, and the motion‐capture data were used only for receiving breathing parameter data by optoelectronic plethysmography.

To eliminate the possibility of voluntary or involuntary effort during tSCS, participants were given a cognitive distraction task that involved silently subtracting a two‐digit number from a four‐digit number in all experimental conditions (Woollacott & Shumway‐Cook, 2002).

### 2.3 Transcutaneous stimulation of the spinal cord

Stimulation was delivered using a Neostim‐5 (LTD Cosyma, Moscow, Russia) at a frequency of 20 Hz, with monopolar modulated current pulses (1 ms duration, 5 kHz carrier frequency).

Stimulation cathodes (diameter, 2.5 cm; ValuTrode, Axelgaard Manufacturing Co., Fallbrook, CA, USA) were positioned along the midline of the back over the spinous processes of T11–T12 and L1–L2 (Figure 1b). Two anodes (5 cm × 10 cm; ValuTrode, Axelgaard Manufacturing Co.) were placed symmetrically over the iliac crests.

The timing of stimulation was synchronized with the Qualisys motion‐capture system.

Stimulation intensity was adjusted individually prior to the recording to the highest level that did not induce pain or discomfort, and the stimulation was then maintained for the entire recording period.

### 2.4 Stabilometry

Ground reaction forces were recorded using an AMTI Optima force platform (model OPT400600‐1K‐STT, Advanced Mechanical Technology, Inc., Watertown, MA, USA). The signals were digitized and synchronized with the Qualisys motion‐capture system, then processed in Visual3D software (C‐Motion Inc., Germantown, MD, USA) for further analysis. The data were sampled at 1000 Hz and filtered using a fourth‐order Butterworth filter with a cut‐off frequency of 10 Hz prior to analysis.

Three parameters were analysed: (1) the 95% confidence ellipse area (ellipse area), computed using the covariance matrix of CoP displacements in the mediolateral (ML) and anteroposterior (AP) directions (Prieto et al., 1996); (2) ML sway, calculated as the root mean square deviation of the CoP in the ML direction (RMSD_ML_); and (3) AP sway, calculated as the root mean square deviation of the CoP in the AP direction (RMSD_AP_).

The effect sizes (*r*) for RMSD_ML_ in control, T11 and L1 tSCS conditions were calculated using the *z*‐score derived from the Wilcoxon signed‐rank test (Fritz et al., 2012). This approach was selected owing to potential violations of normality assumptions in the RMSD_ML_ data. For comparison with previous research, these effect sizes were interpreted according to standard benchmarks (small, *r* = 0.1; medium, *r* = 0.3; large, *r* = 0.5) (Shamantseva et al., 2023, 2024).

### 2.5 Motion capture

#### 2.5.1 Range of motion

Whole‐body kinematics were recorded using the Qualisys motion‐capture system (Qualisys AB, Gothenburg, Sweden), which was equipped with 10 infrared cameras (Oqus 5+) and operated at a sampling rate of 100 Hz. The mean error of the Qualisys Oqus camera has been claimed to be 0.1 mm (Feng & Max, 2014). A total of 55 retroreflective markers were placed on anatomical landmarks according to the full‐body Istituto Ortopedico Rizzoli (IOR) marker model (IOR Gait Overview, 2025; Leardini et al., 2007). This model includes standardized placements on the head, trunk, pelvis and lower extremities. The marker trajectories were labelled and reconstructed in Qualisys Track Manager before being imported into Visual3D for biomechanical modelling. The kinematic data were low‐pass filtered at 6 Hz using a fourth‐order Butterworth filter. Segmental ranges of motion (ROM) were calculated from 3D joint trajectories in the frontal (ML direction) and sagittal (AP direction) planes using local coordinate systems defined for each body segment. ROMs for left and right lower extremities were averaged.

From 55 markers, 16 markers were selected to quantify respiratory parameters using optoelectronic plethysmography.

#### 2.5.2 Intersegmental coordination and postural strategy

Cross‐correlation analysis was performed using MATLAB R2023a (MathWorks, Inc., Natick, MA, USA) to quantify intersegmental coordination. Temporal displacement signals in the ML and AP directions were extracted from the following pairs of segments: head–trunk, trunk–right hip, trunk–left hip, right hip–right ankle and left hip–left ankle. Linear trends were removed from each signal pair, and the cross‐correlation function was computed using a symmetric ± 100‐sample window (±1 s at a sampling rate of 100 Hz). The resulting functions were averaged to obtain a cross‐correlation function for each participant. From this averaged function, the maximum absolute correlation coefficient and its corresponding lag (in seconds) were identified.

According to Winter et al. (1996), balance control in the AP direction during quiet side‐by‐side stance is dependent on ankle plantarflexion/dorsiflexion and is referred to as the ‘ankle strategy’, whereas ML balance is dependent on hip adduction/abduction and is referred to as the ‘hip strategy’. Thus, the cross‐correlation analysis was conducted between CoP displacement in the AP direction and ankle (left and right) motion in the AP direction, and between CoP displacement in the ML direction and hip (left and right) motion in the ML direction.

#### 2.5.3 Optoelectronic plethysmography

The inspiration time (*T*_in_) and expiration time (*T*_ex_) were determined using the previously described method (Shamantseva et al., 2023). The breathing rate (BR) was calculated as 60/(*T*_in_ + *T*_ex_). All resulting time series were processed using fourth‐order Butterworth filters, applying both low‐pass filtering at 1 Hz and high‐pass filtering at 0.08 Hz, followed by normalization. An integrated respiratory waveform was then derived using principal component analysis to combine all measured respiratory signals into a single representative curve that shows the integrated movement of the chest wall and abdomen (Shamantseva et al., 2023).

### 2.6 Electromyography

Surface EMG data were recorded bilaterally over the following muscles: tibialis anterior (TA), soleus (SOL), medial gastrocnemius (GM), rectus femoris (RF), biceps femoris (BF) and vastus lateralis (VL), using a Delsys Trigno wireless system (Delsys Inc., Natick, MA, USA). EMG recording was synchronized with the Qualisys motion‐capture system. The EMG data were sampled at 2148 Hz and processed offline using MATLAB R2023a. The raw EMG signals were band‐pass filtered between 60 and 999 Hz using a second‐order zero‐phase Butterworth filter. The filtered signals were then full‐wave rectified, and the average muscle activity was calculated during two 30 s episodes of quiet standing. For trials involving tSCS, segments surrounding stimulation artefacts were identified based on stimulation peak intervals and excluded from baseline correction and amplitude analysis to minimize contamination. Final EMG amplitudes were obtained by averaging activity within each episode, followed by averaging across the left and right legs.

The co‐activation index (CI) was calculated for the GM–TA, SOL–TA and BF–RF muscle pairs, based on the previously described approach (Moshonkina et al., 2021) using a modified version of the formula by Rudolph et al. (2000).

### 2.7 Data analysis

To ensure signal stability, the first 30 s and final 10 s of the 100 s recording epoch were excluded from analysis. The remaining 60 s were divided into two 30 s episodes, which were analysed separately, then averaged to correspond to our previous studies (Shamantseva et al., 2023, 2024).

To quantify posture–respiratory coupling, cross‐correlation analysis was performed between the respiratory curve and the CoP displacement in the AP direction. Signal epoch was detrended and *z*‐score normalized. The CoP signal was resampled to match the respiratory signal length. A second‐order low‐pass Butterworth filter with a 10 Hz cut‐off was applied to the resampled CoP signal. The normalized respiratory signal and filtered CoP signal were cross‐correlated using a window of ±100 samples (corresponding to ±1 s at 100 Hz). The cross‐correlation function was calculated in the same way as the cross‐correlation between the segments.

Statistical analysis was performed using Analyse‐it for Microsoft Excel (v.6.15.4, 2024; Microsoft Office 2021). The Shapiro–Wilk test was used to assess the normality of data distribution. For data that followed a normal distribution, results are presented as the mean ± SD, and Student's paired *t*‐tests were used for within‐subject comparisons. For non‐normally distributed data, results are presented as the median [first quartile; third quartile], and comparisons were made using the non‐parametric Wilcoxon signed‐rank test. The threshold for statistical significance was set at *p* < 0.05; differences with *p* < 0.1 were interpreted as a trend (Greenland et al., 2016).

## 3 RESULTS

Twenty‐four subjects were recruited, of whom 20 met the inclusion criteria (11 males and nine females, 24 ± 4 years of age). The body mass index of participants was 21.9 ± 1.9 kg/m^2^. Tiffeneau–Pinelli index was 91% ± 6%. All participants considered themselves to be healthy on the day of the study. T11 tSCS intensity was 35 ± 9 mA and L1 tSCS intensity was 34 ± 10 mA for all the participants.

Eleven participants had Gottschaldt coefficients < 2.5 and were classified as FD. Nine participants had Gottschaldt coefficients > 2.5 and were classified as FI (Table 2). There were no significant differences in body mass index or T11 and L1 tSCS intensity between the FD and FI groups (*p* > 0.05).

**TABLE 2:** Base records of the groups of field‐dependent and field‐independent participants.

Group	Sex (male/female)	Gottschaldt coefficient	BMI (kg/m^2^)	T11 (mA)	L1 (mA)
FD (*n* = 11)	6/5	1.7 ± 0.3	20.8 [20.3;24.3]	38 ± 4	38 ± 4
FI (*n* = 9)	5/4	3.3 ± 0.4	21.4 [19.8;23.9]	30 ± 2	29 ± 2

Abbreviations: BMI, body mass index; FD, field dependent; FI, field independent; *n*, number of participants; T11 (L1), current intensity at the T11 (L1) vertebral level.

In the standing position, males had greater SOL activity and T_in_ than females (*p* ≤ 0.01). There were no significant differences in all other analysed parameters in either position. T11 and L1 tSCS did not affect the studied parameters when the groups were analysed by sex (*p* > 0.05).

### 3.1 Stabilometry

**FIGURE 2:**
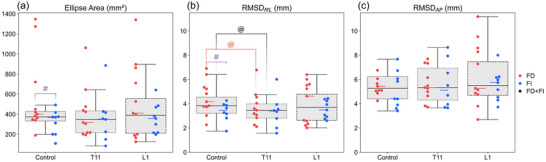
Stabilometry results. Confidence ellipse area of centre of pressure displacements (ellipse area) (a), mediolateral (ML) and anteroposterior (AP) sway as the root mean square deviation of the centre of pressure [RMSD*_ML_* (b) and RMSD*_AP_* (c)] in control conditions and with stimulation at T11–T12 and L1–L2 vertebral levels in all participants (grey boxes), the field‐dependent (FD) group (red circles) and the field‐independent (FI) group (blue circles). ^#^ *p* ≤ 0.08, comparison between FD and FI groups; ^@^ *p *< 0.05, comparison between transcutaneous spinal cord stimulation and control.

The median ellipse area for all the participants was ∼400 mm^2^. FD participants tended to have a greater ellipse area than FI participants (by 8%, *p* = 0.08; Figure 2a; Table S1). No significant differences were found in ellipse area during T11 and L1 tSCS for the combined group of participants or for the FD and FI groups separately.

The median RMSD_ML_ was 4 mm for the combined group. The RMSD_ML_ was 20% greater in the FD group compared with the FI group (*p* = 0.05) in control conditions (Figure 2b; Table S1). During T11 tSCS, RMSD_ML_ decreased significantly in the combined group by 11% (*p* = 0.02) and in the FD group by 21% (*p* = 0.01). This change was associated with a very large effect size (*r* = 0.902) in the FD group, indicating significant decrease in ML sway. L1 tSCS did not significantly affect postural parameters in any group (Figure 2b; Table S1).

The median RMSD_AP_ was 5 mm for all participants (Figure 2c; Table S1). There was no difference in RMSD_AP_ between the FD and FI groups. Neither the combined group nor FD and FI groups showed significant changes during T11 and L1 tSCS.

### 3.2 Muscle activity

#### 3.2.1 Standing

**FIGURE 3:**
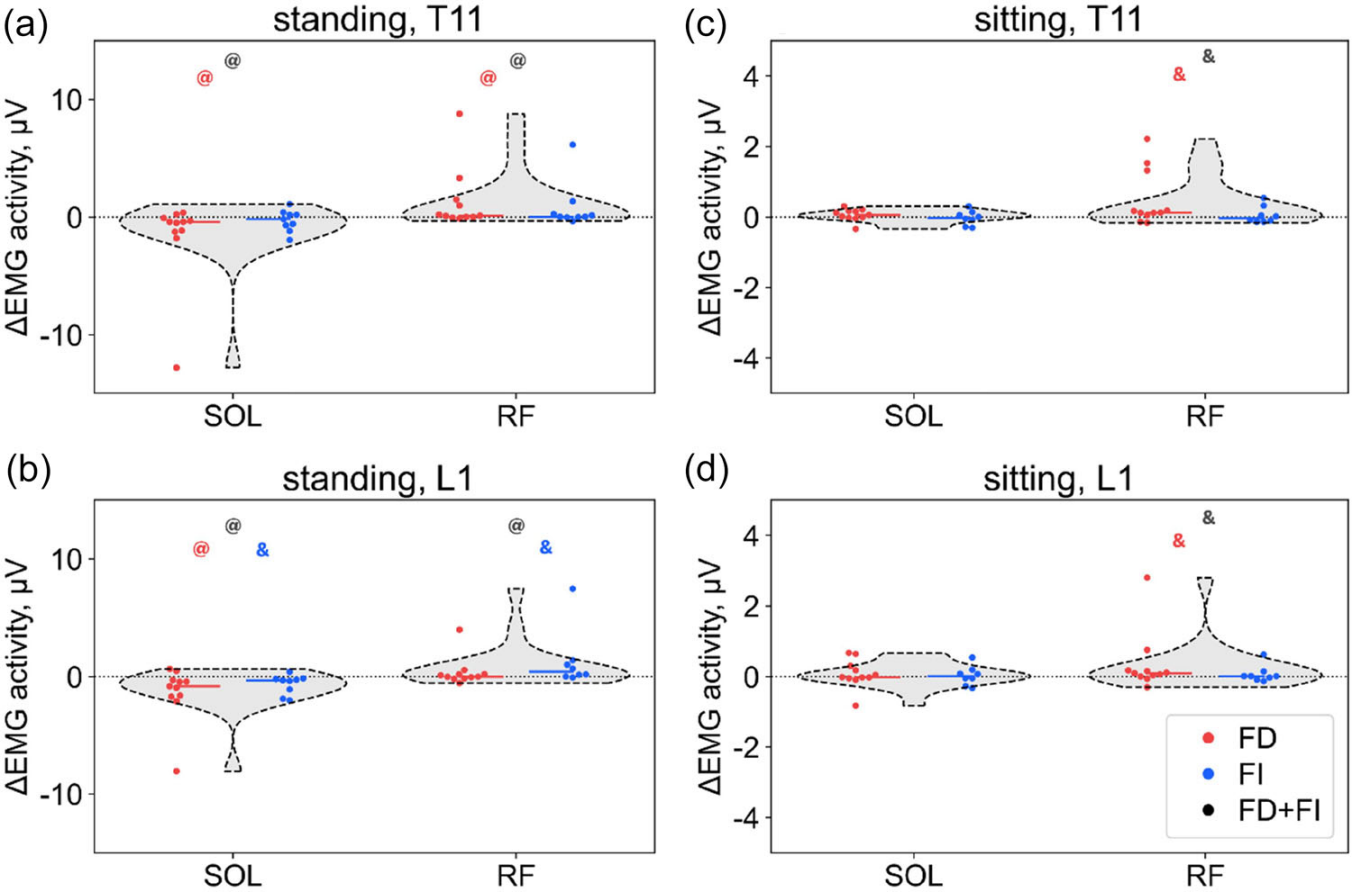
Difference in activity of the soleus (SOL) and rectus femoris (RF) muscles between transcutaneous spinal cord stimulation and control conditions during standing (a, b) and sitting (c, d) for the combined group (grey violins), the field‐dependent (FD) group (red circles) and the field‐independent (FI) group (blue circles); ^@^ *p *< 0.05, ^&^ *p* ≤ 0.07, comparison between control and transcutaneous spinal cord stimulation.

The SOL activity was 6 µV in the combined group during control standing, and there was no difference between the FD and FI groups (Table S2). During T11 stimulation, SOL activity decreased significantly by 5% in the combined group (*p* = 0.01) and by 13% in the FD group (*p* = 0.01) (Figure 3a; Table S2). During L1 stimulation, SOL activity decreased by 11% in the combined group (*p* < 0.01), by 12% in the FD group (*p* = 0.03) and by 9% in the FI group (*p* = 0.05).

The TA activity was 2 µV in the combined group during standing without tSCS (Table S2). FD participants had 25% lower TA activity than FI participants (*p* = 0.04). No significant changes in TA activity occurred during T11 and L1 tSCS.

The GM–TA and SOL–TA CI were 0.78 and 0.88 in the combined group during control standing and were ∼4% lower during T11 and L1 tSCS (*p* = 0.06 and *p* = 0.04, accordingly; Table S3). No changes occurred in GM–TA and SOL–TA CI during T11 tSCS in the FD and FI groups. During L1 stimulation, GM–TA CI was 6% lower in the FD group (*p *< 0.01), and SOL–TA CI was 3% lower in the FD group (*p* = 0.04) and 6% lower in the FI group (*p* = 0.05).

The RF activity was 2 µV in the combined group in control conditions, and there was no difference in RF activity between the FD and FI participants (Table S2). During T11 stimulation, RF activity increased significantly by 4% in the combined group (*p* = 0.01) and by 5% in the FD group (*p* = 0.04) (Figure 3a). During L1 stimulation, RF activity increased by 9% in the combined group (*p* = 0.02) and by 9% in the FI group (*p* = 0.05).

There was no difference in GM, BF and VL activity or in GM–TA, SOL–TA and RF–BF CI between the FD and FI groups in control standing (Table S3). The GM, BF and VL muscles did not react to tSCS (Table S2).

The BF–RF CI was 1.0 in the combined group during control standing and was 5% lower during L1 tSCS (*p* = 0.05; Table S3). In the FI group, this CI was 2% lower during T11 tSCS (*p* = 0.03) and 8% lower during L1 tSCS (*p* < 0.01). No difference occurred for the FD group.

Both T11 and L1 tSCS altered the activity in SOL and RF, and all these changes were followed by a significant decrease in CI.

#### 3.2.2 Sitting

In control conditions, activity of all muscles during sitting was significantly lower than during standing (*p *< 0.01; Table S2), whereas all three CI were greater in sitting compared with standing (*p *< 0.01; Table S3).

The TA activity was 2 µV in the combined group during sitting without stimulation. FD participants had 25% lower TA activity than FI participants (*p* = 0.02; Table S2). There were no significant changes in TA activity during T11 and L1 tSCS.

The SOL–TA CI was 1.1 in the combined group during control sitting and decreased by 7% during T11 tSCS (*p* < 0.01) and by 5% during L1 tSCS (*p* = 0.01) (Table S3). In the FD group, this CI decreased by 6% during T11 tSCS (*p* < 0.01) and by 5% during L1 tSCS (*p* = 0.06). There were no changes in the FI group. There was no difference in SOL–TA, GM–TA and BF–RF CI between the FD and FI groups, and GM–TA and BF–RF CI did not change during tSCS.

The RF activity was 2 µV in the combined group in control conditions, and there was no difference in RF activity between the FD and FI participants (Table S2). During T11 stimulation, RF activity increased by 6% in the combined group (*p* = 0.07) and by 7% in the FD group (*p* = 0.05; Figure 3b). During L1 stimulation, RF activity increased by 2% in the combined group (*p* = 0.07) and by 6% in the FD group (*p* = 0.05).

### 3.3 Kinematics of the body during standing

#### 3.3.1 Range of motion

**FIGURE 4:**
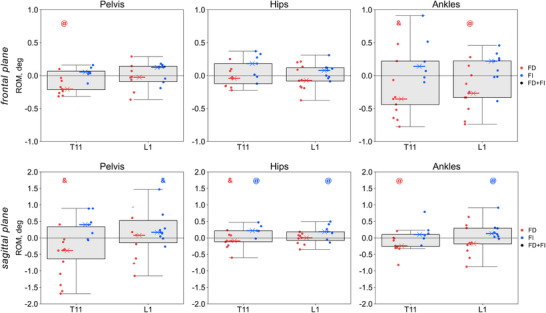
Difference in the range of motion (ROM) between control standing and transcutaneous spinal cord stimulation (tSCS) for the combined group (grey boxes), the field‐dependent (FD) group (red circles) and the field‐independent (FI) group (blue circles). Results for frontal and sagittal planes are presented (ROM in the mediolateral and anteroposterior directions, respectively). Outliers are not shown; ^@^ *p *< 0.05 comparison between tSCS and control, ^&^ *p* ≤ 0.07 comparison between tSCS and control.

In the frontal plane, Pelvis_ML_ ROM was 18% greater in the FD group than in the FI group (*p* = 0.04), and Ankle_ML_ ROM also tended to be greater in the FD group (*p* = 0.07; Figure 4; Table S4). During T11 tSCS, Pelvis_ML_ ROM decreased significantly by 35% only in the FD group (*p* = 0.03), along with a trend towards a decrease in the Ankle_ML_ ROM (*p* = 0.07). In contrast, the FI participants showed a significant 10% increase in Trunk_ML_. During L1 tSCS, Ankle_ML_ ROM decreased significantly by 17% in the FD group, whereas no significant changes in frontal ROM were observed in the FI group.

In the sagittal plane, Pelvis_AP_ was 32% greater in the FD group than in the FI group (*p* = 0.05; Figure 4; Table S4). During T11 tSCS, Pelvis_AP_ ROM decreased by 20% in the FD group (*p* = 0.05), followed by significant decreases in the Knee_AP_ ROM (21%, *p* = 0.03) and Ankle_AP_ ROM (24%, *p* = 0.03) and a tendency towards a decrease in Hip_AP_ ROM (17%, *p* = 0.07). Meanwhile, Hip_AP_ ROM increased by 40% in the FI group (*p* < 0.01) during T11 tSCS. During L1 tSCS, a significant increase occurred in the Hip_AP_ ROM (31%, *p* = 0.01) and Ankle_AP_ ROM (16%, *p *< 0.01) in the FI group. L1 tSCS did not affect sagittal ROM for the FD participants.

#### 3.3.2 Intersegmental coordination

Frontal coordination between the head and trunk was 0.5 with no lag for the combined group and FD participants; FI participants had slightly lower head–trunk_ML_ coordination with a coefficient of 0.4 and no lag (Table S5). There was no significant correlation (≤0.2) between the trunk and both hips in the ML direction in either group. The right hip and right ankle coordination was significantly stronger for the FD participants during control standing, with cross‐correlation coefficient of −0.5 with no lag, compared to 0.2 with −0.2 s lag for the FI group. During T11 tSCS, only head–trunk_ML_ coordination tended to increase by 10% in the FD group (*p* = 0.06), with no changes observed in the FI group. No significant changes in frontal intersegmental coordination were found during L1 tSCS in either group.

Sagittal coordination between the head and trunk was 0.2 with −0.08 s lag for the combined group and FD participants, which indicates weak coupling between the two segments (Table S5). FI participants had slightly greater head‐trunk_AP_ coordination of 0.4 with −0.03 s lag. There was no significant correlation (≤0.2) between the trunk and both hips in the AP direction in either group. Overall, intersegmental coordination in the AP direction did not differ between FD and FI participants during control standing. No significant changes were observed in either group during T11 tSCS. During L1 tSCS, trunk–left hip AP coordination increased significantly by 25% (*p* < 0.01), and trunk–right hip AP coordination showed a trend towards a 20% increase (*p* = 0.06) in the FD group. No significant effects of L1 tSCS were observed in the FI group.

In control standing, head–trunk and both hip–ankle segments showed stronger coordination in the ML direction than in the AP direction, primarily owing to higher correlation values observed in the FD group.

#### 3.3.3 CoP‐segment coordination patterns

In the ML direction, the right hip and CoP displacement had anti‐phase coordination in the combined group (−0.5 with −0.18 s lag; Table S6). This coordination was more pronounced in the FD group, with a cross‐correlation coefficient of −0.6 with −0.18 s lag, in comparison to the FI group (−0.4 at −0.17 s; *p* = 0.06). The left hip and CoP displacement showed strong in‐phase coordination in the combined group, with a coefficient of 0.5 with −0.07 s lag, that was also attributable to the greater correlation for the FD participants (0.6 with −0.06 s lag) compared with the FI participants (0.3 at −0.09 s; *p* = 0.06). This indicates that both hips followed CoP shifts predominantly in the FD group. T11 and L1 tSCS did not affect these coordination patterns in either group.

In the AP direction, both ankles demonstrated similarly strong anti‐phase coordination with CoP displacement across all participants (correlation coefficient = −0.7, with no lag; Table S6). This pattern remained stable during both T11 and L1 stimulation and was unaffected by cognitive style.

### 3.4 Respiration

#### 3.4.1 Standing

The BR was 17 breaths/min, *T*_in_ 1.3 s and *T*_ex_ 2.1 s for all the participants in control standing (Table S7). There was no difference in breathing parameters between the FD and FI participants. No significant changes occurred during T11 and L1 tSCS.

#### 3.4.2 Sitting

The BR was 18 breaths/min, *T*_in_ 1.3 s and *T*_ex_ 1.9 s for all the participants in control sitting (Table S7). There was no difference in breathing parameters between the FD and FI participants. No significant changes occurred during T11 and L1 tSCS.

#### 3.4.3 CoP–respiratory coupling

**FIGURE 5:**
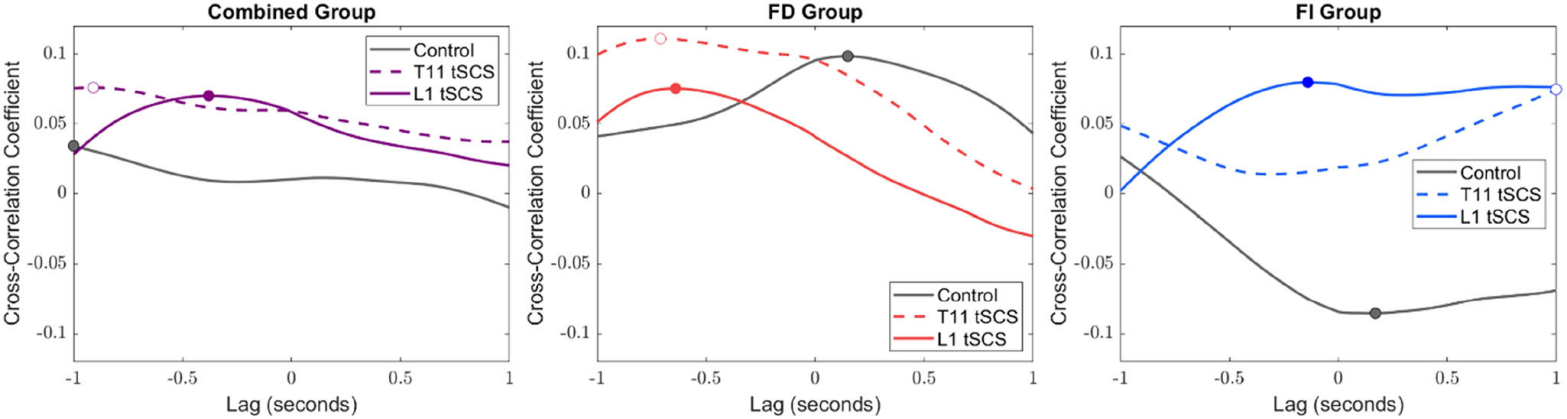
Averaged cross‐correlation functions and corresponding lags between respiratory curve and centre of pressure displacement in the mediolateral direction in the combined group, in the field‐dependent (FD) group and in the field‐independent (FI) group in control, T11 and L1 transcutaneous spinal cord stimulation (tSCS) standing. The lag corresponding to the peak correlation is highlighted as a circle for each cross‐correlation function.

Posture–respiratory synchronization did not occur for the combined group during control standing or for the FD and FI groups separately; the cross‐correlation coefficient was ≤ ±0.1 in ML (Figure 5) and AP (Figure S1) directions. T11 and L1 tSCS did not induce posture–respiratory coupling.

## 4 DISCUSSION

### 4.1 Quiet standing without stimulation

In control standing with eyes and ears closed, all the participants exhibited low postural sway and minimal joint motion. All registered muscle activity was greater in the standing position than in the sitting position, confirming previous evidence that postural muscles, such as the SOL and GM, are more engaged during upright stance to maintain balance (Zellers et al., 2020). The CI was significantly lower in standing compared with sitting, indicating that agonist and antagonist muscles had a more equal and generalized activation in the seated position (Rudolph et al., 2000). During standing, SOL and GM activity were greater than BF and RF activity (Table S2). This observation is consistent with the previous findings of distal‐to‐proximal EMG activity for slow transitions that induce corrective responses, which begin with the posterior muscles of the lower limbs (Horak et al., 1990; Runge et al., 1999). Together, these neuromuscular activation patterns confirm that the collected data are consistent with established physiological norms and support the reliability of our experimental approach.

The ROM in joint angles showed greater motion at trunk_AP_, head_AP_ and head_ML_ segments (Table S4). There was weak coordination between the ankles and hips in the AP direction and moderate coordination in the ML direction (Table S5). Ankle_AP_ motion showed strong anti‐phase correlation with CoP displacement in the AP direction, with a lag around zero (Table S6). Hip_ML_ showed moderate correlation with CoP displacement in the ML direction. The correlation was synphase with a lag ∼100 ms on the side of the supporting leg and antiphase with a lag ∼200 ms on the side of the dominant leg. The results confirm the previously described strategy for postural control during normal quiet stance; the ankle mechanism dominates in the sagittal plane and the hip mechanism follows in the frontal plane (Gatev et al., 1999).

Posture–respiratory synchronization was absent in our group of healthy young participants. This aligns with the idea that respiration‐induced sway is suppressed in healthy participants (Manor et al., 2012). There was no difference in posture–respiratory coupling between FD and FI individuals, indicating that cognitive style does not affect this compensatory postural mechanism.

### 4.2 Group differences (FD vs. FI)

Previous studies have shown a close relationship between interindividual variability in both perception of external space and postural control (Isableu et al., 2003). FD people find it difficult to maintain a vertical posture without any external sensory information. Previous studies have documented the peculiarities of CoP displacement and body segment coordination in FD and FI individuals when standing quietly in the absence of visual and audio cues (Isableu et al., 2003, 2010; Shamantseva et al., 2023, 2024). We essentially replicated these findings. Additionally, we characterized the peculiarities of muscle activity and sagittal body kinematics in these groups.

FD individuals exhibited greater ML sway of CoP, with larger pelvis_ML_ ROM, significant hip–ankle frontal coordination and reduced TA activity compared with FI participants. In contrast, FI participants demonstrated tighter postural control, with smaller pelvis and trunk motion and greater activation of TA. There was no significant difference in sagittal intersegmental coordination between the FD and FI groups.

Previous research on intersegmental differences in the frontal plane between participants with pronounced FD and FI was conducted in conditions of gradual visual distortion and increasing stance difficulty (individuals stood with feet together or a heel‐to‐toe posture) (Isableu et al., 2003, 2010). Isableu and colleagues demonstrated that participants with pronounced FD used ‘*en bloc*’ head–shoulder or shoulder–hip coordination in total darkness, which extended to hip stabilization strategies when difficulty of stance increased. In contrast, participants with pronounced FI tended to move their head, trunk and hip segments independently, displaying no ‘*en bloc*’ pattern across conditions. In our study, we observed a moderate positive frontal coordination between head and trunk motion and weak trunk–hip coordination in ML and AP directions in both groups. The differences we observed are associated with the consistent normal stance (heels together and toes apart) that we examined in our study.

In terms of postural strategies, both groups showed strong anti‐phase correlations between ankle motion and CoP displacement in the AP direction. However, FD participants demonstrated a greater correlation between hip motion and ML CoP displacement than FI participants, suggesting a shift away from pure ankle strategy. Thus, both the presence and the absence of external sensory information influence individual differences in intersegmental coordination, as evidenced by the fact that in identical sensory conditions (with vision and hearing blocked), FD and FI individuals still adopted distinct postural coordination patterns. Taking this factor into account in models of postural control strategies (such as the ‘inverted pendulum’ and others) will make them more accurate.

### 4.3 Respiratory effects of tSCS

T11 and L1 tSCS produced no changes in breathing parameters in either sitting or standing conditions. In contrast, previous studies have reported an increase in BR and decrease in tidal volume during T11 tSCS while participants were in a side‐lying position (Minyaeva et al., 2017, 2019). The authors suggested that one of the possible reasons for the respiratory response might be the direct stimulation of abdominal muscles with bipolar electrical pulses (Minyaeva et al., 2019). The cathode was placed on the back, and a couple of anodes were placed on the front surface of the body, above the iliac crests. In the case of bipolar pulses, the function of the back and front electrodes changes during a pulse. The front electrodes are anodes during one half of the pulse and cathodes during the other. Thus, direct muscle stimulation is possible. In our study, tSCS was applied using monopolar pulses, and it was not possible to stimulate the abdominal muscles directly using a similar electrode montage.

Thus, we found no change in external respiratory parameters during lumbar tSCS in the sitting position. This suggests that stimulation with the used parameters does not affect respiratory movements in young, healthy individuals. Likewise, we found no effect of tSCS on respiration in the standing position, whether or not tSCS affected postural stability. Consequently, all effects of tSCS on postural stability are solely associated with modulation of spinal posture‐regulation networks.

### 4.4 Postural effects of tSCS

Several tSCS parameters, including the waveform, intensity and frequency, might have distinct effects on spinal networks, motor pools and corticospinal excitability (Benavides et al., 2020; Massey et al., 2024).

A study on participants with chronic paralysis after spinal cord injury has demonstrated that lumbar tSCS at motor threshold intensity improves postural stability and trunk control (Sayenko et al., 2019). The authors concluded that tSCS has a frequency‐dependent effect on motor output, with stimulation at 15–25 Hz producing a more pronounced effect on self‐assisted standing in comparison to 5 Hz. They further suggested that tSCS enhances the excitability of posture‐specific interneuronal networks during the transition to standing (Sayenko et al., 2019). Our results confirm this conclusion. We observed a decrease in the activity of the postural muscle (SOL) when we stimulated the thoracolumbar region and increased postural stability (Figure 2). Thus, rather than activating the muscles directly, tSCS might have influenced the interneuronal spinal networks responsible for the redistribution of muscle activity to control postural stability. It should also be noted that we used tSCS intensities at the level of tolerance, which are lower than those that elicit a direct muscle response (Manson et al., 2020; Yang et al., 2025).

Studies on healthy volunteers have demonstrated that postural effects of lumbar tSCS are segment specific (Carrera et al., 2022; Omofuma et al., 2024; Shamantseva et al., 2023, 2024, 2024). In a stance disturbed by trunk perturbation, tSCS applied between the L2 and L3 vertebrae (5–10 mA, 30 Hz) reduced postural stability in the AP direction (Carrera et al., 2022; Omofuma et al., 2024). During quiet standing in a soundproof chamber with the eyes closed, tSCS applied between the L1 and L2 vertebrae (12–36 mA, 20 Hz) increased postural sway in the AP direction, but only in the FD participants (Shamantseva et al., 2023). In similar conditions, tSCS applied to the T11–T12 vertebral level (19–43 mA, 20 Hz) improved postural stability and decreased postural sway in the ML direction in the FD participants (Shamantseva et al., 2024). When combined with destabilizing auditory stimuli, only T11–T12 tSCS (23–39 mA, 20 Hz) stabilized posture, presumably via the activation of spinal networks at the supraspinal level, and no postural effects occurred with L1–L2 tSCS (20–36 mA, 20 Hz) (Shamantseva et al., 2024). All these cited studies used stimulation parameters (frequency and intensity) that specifically activated spinal posture regulation interneurons rather than directly activating postural muscles.

#### 4.4.1 T11 tSCS

The present results show that T11 tSCS led to significant decrease in ML sway in the combined group, driven primarily by a 21% decrease in the FD group. This stabilizing effect was accompanied by reduced SOL activity, increased RF activity and significantly decreased ROM in the pelvis_ML_, knee_AP_ and ankle_AP_ segments, followed by a trend of decrease in the pelvis_AP_, hip_AP_ and ankle_ML_ in the FD individuals. T11 tSCS further increased head–trunk coordination in the ML direction, leaving no change in postural strategy for the FD participants. These findings partly support our previous hypothesis that the stabilizing effect of T11 tSCS is achieved via selective recruitment of hip flexors that leads to hip stabilization (Shamantseva et al., 2024).

In contrast, FI participants did not exhibit a change of CoP parameters during T11 tSCS, and in these participants no redistribution of muscle activity during stimulation was recorded. Instead, they showed an increase of trunk_ML_ and hip_AP_ ROM with a decrease in the BF–RF CI and no change in intersegmental coordination. For FI participants, the absence of visual and auditory cues does not destabilize posture (Shamantseva et al., 2023, 2024), and activation of spinal postural networks does not lead to measurable changes in postural sway. This response reflects different strategies of postural control in FD and FI individuals during standing without visual and audio information, when the former suppress segmental motion to increase stability, whereas the latter adapt by modulating segmental degrees of freedom.

#### 4.4.2 L1 tSCS

L1 tSCS produced no significant change in postural parameters in the combined group or in the FD and FI individuals. Nevertheless, we recorded significant changes in muscle activity and segmental ROM. In the FD group, a decrease in SOL activity and in SOL–TA and GM–TA CIs was observed. This decrease was combined with a reduction in ankle_ML_ ROM. In FI participants, an increase in hip_AP_ and ankle_AP_ ROM and decrease in BF–RF CI were observed. These changes did not affect intersegmental coordination in either group.

Taken together, these results do not support our previous hypothesis that L1 tSCS increases postural instability via increasing ankle joint stiffness (Shamantseva et al., 2023). That hypothesis was based on a prior study in which L1 tSCS was applied during the stance phase of walking and resulted in an increase of ∼10% in CI for the BF–VL and ∼5% for the GM–TA muscle pairs (Moshonkina et al., 2021). In contrast, CI values observed in the present study during quiet standing (Table S3) were three to four times higher than those recorded during the stance phase and significantly decreased during L1 tSCS, opposite to the previously reported increase (Moshonkina et al., 2021). The ∼5% reduction in GM–TA CI obtained in the present study did not affect postural stability. The opposite response to the similar stimulation highlights the significance of afferent information in the functioning of spinal motor centres.

Interestingly, although L1 tSCS alone has previously been shown to increase postural sway significantly, particularly in FD individuals (Shamantseva et al., 2023) this destabilizing effect appears to be mitigated when L1 stimulation is combined with or preceded by T11 tSCS in the study protocol. Our recent study found that applying T11 tSCS during affective auditory stimulation reduced lateral sway when no postural effect occurred during L1 tSCS (Shamantseva et al., 2024). In the present study, half of the participants started with T11 tSCS and the other half with L1 tSCS. Thus, 5 of 11 FD participants and 5 of 9 FI participants started the experimental procedures with L1 tSCS. Of these, 3 of 5 FD participants and 4 of 5 FI participants experienced an increase in the ellipse area during L1 tSCS in comparison to the conditions with no stimulation. If the spinal interneurons activated at the L1–L2 vertebral level are indeed sensitive to preceding upper‐level tSCS, it is noteworthy that the networks at the T11–T12 level produce consistent effects regardless of prior lower‐level stimulation.

Based on these findings, we suggest that spinal neural networks at the T11–T12 level are critically involved in stabilizing upright posture in response to destabilizing supraspinal input, possibly by integrating descending affective or sensorimotor signals. In our study, the absence of visual and auditory information served as such a destabilizing supraspinal signal. In contrast, the L1–L2 networks appear less involved in this regulation (Shamantseva et al., 2024). The present results are consistent with this interpretation, because only T11 tSCS led to a significant reduction in ML sway. Thus, the modulatory effects of tSCS on postural control occurred within the physiological range. Consequently, the conclusions drawn from these results contribute to our understanding of spinal mechanisms that underlie postural regulation in normal physiological conditions.

### 4.5 Limitations

In this study, participants performed tasks with their eyes covered and ears plugged. Although this approach effectively isolated somatosensory contributions to postural control, it constitutes an artificial condition that might limit the generalizability of our findings to everyday balance situations. T11 and L1 tSCS were applied within the same session, which might have resulted in them influencing each other. Using separate‐day stimulation protocols might help to minimize such effects.

## 5 CONCLUSION

Previous studies have described differences in frontal plane body segment movements between FD and FI individuals during unstable stance conditions without visual or auditory input. Our findings extend these observations to normal stance conditions, revealing that although FD and FI participants exhibited similar segmental coordination patterns in the sagittal plane, they demonstrated distinct coordination strategies in the frontal plane. Taking this into account when developing models of postural control strategies will improve their accuracy.

Previous studies suggest that interneuronal networks involved in postural regulation might be influenced by tSCS applied at the T11–T12 vertebral level at subthreshold motor intensities. Our findings are consistent with prior works, because the observed postural modulations align with the proposed effects of tSCS on spinal networks at this anatomical level.

Stimulation of the spinal cord at the T11–T12 vertebral level induces distinct changes in leg muscle activity and body segment coordination in FD and FI individuals. In FD individuals, such stimulation leads to a stabilization of upright posture, whereas in FI individuals, it does not alter postural sway. These differences are likely to reflect distinct postural control strategies and the varying influence of supraspinal input in the absence of external sensory cues.

Activation of interneuronal networks at the L1–L2 level also results in redistribution of leg muscle activity and changes in body segment coordination, but without measurable effects on postural stability. The differential responses observed between FD and FI individuals suggest that these networks might be subject to supraspinal influences, although their specific functional role in postural regulation requires further investigation.

## AUTHOR CONTRIBUTIONS

Tatiana Moshonkina: Conceptualization, methodology, supervision, writing—review, and editing. Natalia Shamantseva: Conceptualization, methodology, data curation, formal analysis, investigation, writing—original draft, and visualization. Vsevolod Lyakhovetskii: Validation, data curation, formal analysis, writing—review, and editing. Andrey Aksenov: Methodology, validation, data curation, and formal analysis. Tatiana Klishkovskaia: Data curation, formal analysis. Ivan Sakun: Investigation, data curation. All authors approved the final version of the manuscript and agree to be accountable for all aspects of the work in ensuring that questions related to the accuracy or integrity of any part of the work are appropriately investigated and resolved. All persons designated as authors qualify for authorship, and all those who qualify for authorship are listed.

## CONFLICT OF INTEREST

Tatiana Moshonkina is a researcher on the study team and holds shareholder interest in Cosyma Ltd (Moscow, Russia). She holds certain inventorship rights on intellectual property licensed by Cosyma. The remaining authors declare no conflicts of interest.

## Supporting information



Table S1: Center of pressure parameters in control, T11 tSCS and L1 tSCS
Table S2: EMG activity (µV) for both legs in control, T11 tSCS and L1 tSCS in standing and sitting positions
Table S3: Coactivation indices in control and tSCS conditions for FD and FI groups in standing and sitting positions
Table S4: Range of motion (degrees) in control, T11 and L1 tSCS for FD and FI groups in standing position
Table S5: Intersegmental cross‐correlation coefficients (CC) and corresponding lags (in seconds) in control, T11 and L1 tSCS for the FD and FI groups in standing position
Table S6: Cross‐correlation coefficients (CC) and corresponding lags (in seconds) between hips and CoP frontal motion and between ankles and CoP sagittal motion in control, T11 and L1 tSCS for the FD and FI groups in standing position
Table S7: Respiratory parameters in control and tSCS conditions for FD and FI groups in sitting and standing positions
Figure S1: Averaged cross‐correlation functions and corresponding lags between respiratory curve and CoP displacement in the AP direction in the combined group, in the FD group and in the FI group in control, T11 and L1 tSCS standing.

## Data Availability

The datasets analysed in the present study are available from the corresponding author upon reasonable request.
