# Supplementary material for: Postural control in humans: a study using transcutaneous spinal cord stimulation
Source: Exp Physiol. 2025 Nov 22;111(4):2163–75. doi: 10.1113/EP093385 (PMC13140641; doi:10.1113/EP093385)
Supplement: Supplementary file 1 — Table S1: Center of pressure parameters in control, T11 tSCS and L1 tSCS Table S2: EMG activity (µV) for both legs in control, T11 tSCS and L1 tSCS in standing and sitting positions Table S3: Coactivation indices in control and tSCS conditions for FD and FI groups in standing and sitting positions Table S4: Range of motion (degrees) in control, T11 and L1 tSCS for FD and FI groups in standing position Table S5: Intersegmental cross‐correlation coefficients (CC) and corresponding lags (in seconds) in control, T11 and L1 tSCS for the FD and FI groups in standing position Table S6: Cross‐correlation coefficients (CC) and corresponding lags (in seconds) between hips and CoP frontal motion and between ankles and CoP sagittal motion in control, T11 and L1 tSCS for the FD and FI groups in standing position Table S7: Respiratory parameters in control and tSCS conditions for FD and FI groups in sitting and standing positions Figure S1: Averaged cross‐correlation functions and corresponding lags between respiratory curve and CoP displacement in the AP direction in the combined group, in the FD group and in the FI group in control, T11 and L1 tSCS standing. [file EPH-111-2163-s001.docx]

**Table S1.** Center of pressure parameters in control, T11 tSCS and L1 tSCS.

|  | **Control** | | | **T11** | | | **L1** | | |
| --- | --- | --- | --- | --- | --- | --- | --- | --- | --- |
|  | FD+FI | FD | FI | FD+FI | FD | FI | FD+FI | FD | FI |
| Ellipse area, mm^2^ | 374 [333;427] | 400 [354;585]^#^ | 369 [200;376] | 348 [216;436] | 311 [216;466] | 355 [224;424] | 390 [212;556] | 411 [195;786] | 357 [217;469] |
| RMSD_ML_, mm | 3.8 [3.2;4.5] | 4.1 [3.7;5.2]^&^ | 3.4 [3.0;3.8] | 3.4 [2.7;4.0]^@^ | 3.3 [2.9;4.3]^@^ | 3.4 [2.7;3.9] | 3.7 [2.6;4.7] | 4.6 [2.4;5.5] | 3.4 [2.9;4.2] |
| RMSD_AP_, mm | 5.2 [4.4;6.2] | 5.4 [5.0;6.4] | 4.4 [3.9;6.0] | 5.3 [4.0;7.2] | 5.3 [4.7;7.0] | 5.4 [3.9;6.5] | 5.5 [4.7;7.4] | 5.2 [4.8;8.4] | 5.7 [4.5;6.1] |

^&^p = 0.05, ^#^p = 0.08, comparison between FD and FI groups; ^@^p<0.05, comparison between tSCS and control.

**Table S2.** EMG activity (µV) for both legs in control, T11 tSCS and L1 tSCS in standing and sitting positions.

| **Muscle** | **Control** | | | **T11** | | | **L1** | | |
| --- | --- | --- | --- | --- | --- | --- | --- | --- | --- |
|  | FD+FI | FD | FI | FD+FI | FD | FI | FD+FI | FD | FI |
| standing | | | | | | | | | |
| TA | 2.2 [1.8;2.6]^γ^ | 2.1 [1.4;2.3]* | 2.8 [2.0;4.6] | 1.9 [1.7;3.4] | 1.7 [1.5;2.1] | 2.4 [1.7;3.8] | 2.2 [1.7;4.4] | 2.2 [1.5;5.2] | 2.3 [1.9;4.4] |
| GM | 4.1 [3.0;4.8]^γ^ | 3.9 [2.7;5.0] | 4.2 [2.8;5.4] | 3.5 [2.7;5.3] | 3.5 [2.4;4.8] | 3.7 [3.0;6.0] | 3.5 [2.6;5.3] | 3.5 [2.7;4.9] | 3.0 [2.6;7.0] |
| SOL | 5.9 [4.0;10.6]^γ^ | 6.8 [5.4;10.9] | 5.3 [3.1;8.4] | 5.6 [3.6;7.7]^@^ | 6.1 [3.9;9.5]^@^ | 4.9 [3.4;7.5] | 5.1 [3.2;10.3]^@^ | 6.2 [3.4;10.7]^@^ | 4.4 [2.8;7.1]^&^ |
| RF | 2.2 [1.8;2.6]^γ^ | 2.3 [1.7;2.6] | 2.0 [1.7;2.7] | 2.4 [1.8;3.6]^@^ | 2.5 [2.2;3.5]^@^ | 1.9 [1.7;3.3] | 2.5 [1.9;3.0]^@^ | 2.5 [2.4;3.4] | 2.1 [1.8;3.3]^&^ |
| BF | 1.9 [1.6;3.4]^γ^ | 2.2 [1.6;4.1] | 1.9 [1.6;3.3] | 2.3 [2.0;3.1] | 2.8 [2.3;3.8] | 2.0 [1.7;2.5] | 2.2 [1.8;3.0] | 2.6 [1.8;3.7] | 2.1 [1.7;2.5] |
| VL | 2.5 [2.0;3.6]^γ^ | 2.2 [1.8;3.5] | 3.1 [2.2;4.3] | 2.4 [1.8;5.0] | 2.0 [1.8;3.4] | 3.3 [2.1;5.2] | 2.6 [2.1;3.7] | 2.3 [1.7;3.4] | 2.9 [2.4;4.7] |
| sitting | | | | | | | | | |
| TA | 1.7 [1.5;2.2] | 1.6 [1.2;1.9]* | 2.1 [1.7;3.7] | 1.7 [1.5;2.1] | 1.5 [1.2;2.0] | 1.9 [1.7;2.6] | 1.7 [1.6;2.2] | 1.6 [1.2;1.8] | 2.0 [1.7;2.9] |
| GM | 1.9  [1.6;2.0] | 1.9 [1.4;2.0] | 1.9 [1.8;2.2] | 1.8 [1.5;2.0] | 1.6 [1.4;2.0] | 1.8 [1.8;2.0] | 1.8 [1.6;2.0] | 1.7 [1.4;2.0] | 1.8 [1.8;2.0] |
| SOL | 2.0 [1.6;2.5] | 1.6 [1.5;2.3] | 2.2 [1.9;3.0] | 1.8 [1.6;2.5] | 1.8 [1.5;2.4] | 2.0 [1.8;3.1] | 1.9 [1.6;2.7] | 1.7 [1.4;2.1] | 2.2 [1.8;3.2] |
| RF | 1.8 [1.6; 2.2] | 1.8 [1.4;2.2] | 1.9 [1.7;2.2] | 2.0 [1.8;2.7]^&^ | 2.0 [1.6;3.1]^&^ | 2.0 [1.8;2.4] | 1.9 [1.8;2.3]^&^ | 1.9 [1.7;2.6]^&^ | 2.0 [1.8;2.3] |
| BF | 1.5 [1.4;1.7] | 1.4 [1.3;1.8] | 1.6 [1.5;1.6] | 1.6 [1.5;1.8] | 1.8 [1.5;2.1] | 1.6 [1.5;1.6] | 1.6 [1.4;1.8] | 1.7 [1.4;2.0] | 1.5 [1.4;1.6] |
| VL | 1.9 [1.6;2.1] | 1.6 [1.2;1.9]* | 2.1 [1.9;3.2] | 1.8 [1.6;2.0] | 1.7 [1.5;1.9] | 2.2 [1.8;3.3] | 1.8 [1.7;2.0] | 1.7 [1.6;1.9] | 2.0 [1.9;3.4] |

^γ^p<0.01, comparison between control standing and control sitting; ^*^p<0.05, comparison between FD and FI groups; ^@^p<0.05, comparison between tSCS and control, ^&^p≤0.07, comparison between tSCS and control.

**Table S3** Coactivation indices in control and tSCS conditions for FD and FI groups in standing and sitting positions.

| **Pair** | **Control** | | | **T11** | | | **L1** | | |
| --- | --- | --- | --- | --- | --- | --- | --- | --- | --- |
|  | FD+FI | FD | FI | FD+FI | FD | FI | FD+FI | FD | FI |
|  | standing | | | | | | | | |
| GM-TA | 0.78 [0.7;0.8]^γ^ | 0.82 [0.7;0.8] | 0.76 [0.6;0.8] | 0.75 [0.6;0.8]^&^ | 0.77 [0.6;0.8] | 0.74 [0.6;0.7] | 0.76 [0.7;0.8]^@^ | 0.77 [0.7;0.8]^@^ | 0.74 [0.6;0.7] |
| SOL-TA | 0.88 [0.8;0.9]^γ^ | 0.90 [0.8;1.0] | 0.86 [0.7;0.9] | 0.87 [0.7;0.9]^@^ | 0.91 [0.7;0.9] | 0.86 [0.7;0.8] | 0.85 [0.7;0.9]^@^ | 0.88 [0.6;0.9]^@^ | 0.81 [0.7;0.8]^&^ |
| BF-RF | 1.01 [0.9;1.0]^γ^ | 1.00 [0.9;1.0] | 1.02 [1.0;1.0] | 0.97 [0.8;1.0] | 0.96 [0.9;0.9] | 1.00 [0.8;1.0]^@^ | 0.97 [0.9;1.0]^&^ | 0.96 [0.9;1.0] | 0.94 [0.9;1.0]^@^ |
|  | sitting | | | | | | | | |
| GM-TA | 1.08 [1.0;1.1] | 1.07 [1.0;1.1] | 1.09 [1.0;1.1] | 1.07 [1.0;1.1] | 1.06 [1.0;1.1] | 1.07 [1.0;1.1] | 1.05 [1.0;1.1] | 1.07 [1.0;1.1] | 1.04 [1.0;1.1] |
| SOL-TA | 1.10 [1.0;1.1] | 1.08 [1.0;1.1] | 1.10 [1.0;1.1] | 1.02 [0.9;1.1]^@^ | 1.02 [0.9;1.1]^@^ | 1.04 [0.9;1.0] | 1.03 [0.9;1.1]^@^ | 1.02 [0.9;1.1]^&^ | 1.06 [1.0;1.1] |
| BF-RF | 1.14 [1.1;1.1] | 1.13 [1.0;1.1] | 1.15 [1.1;1.1] | 1.11 [1.0;1.1] | 1.11 [1.0;1.1] | 1.12 [1.1;1.1] | 1.10 [1.0;1.1] | 1.09 [1.0;1.1] | 1.13 [1.0;1.1] |

^γ^p<0.01, comparison between control standing and control sitting; ^@^p<0.05, ^&^p≤0.07 comparison between tSCS and control.

**Table S4.** Range of motion (degrees) in control, T11 and L1 tSCS for FD and FI groups in standing position.

| **Segment** | **Control** | | | **T11** | | | **L1** | | |
| --- | --- | --- | --- | --- | --- | --- | --- | --- | --- |
|  | FD+FI | FD | FI | FD+FI | FD | FI | FD+FI | FD | FI |
| Head_AP_ | 3.8 [2.6;6.6] | 3.5 [2.6;7.0] | 4.1 [3.1;6.3] | 3.8 [2.8;7.0] | 3.6 [2.8;4.5] | 6.0 [2.9;9.3] | 3.7 [2.7;5.4] | 3.6 [2.7;4.2] | 3.8 [3.0;5.9] |
| Head_ML_ | 2.1 [1.7;3.5] | 2.9 [1.7;4.6] | 2.0 [1.8;2.2] | 1.9 [1.4;2.3] | 1.5 [1.3;2.1] | 2.1 [1.6;3.0] | 2.1 [1.3;2.5] | 1.7 [1.2;2.1] | 2.4 [1.6;2.6] |
| Trunk_AP_ | 2.0 [1.7;2.7] | 1.9 [1.7;2.8] | 2.0 [1.7;2.7] | 2.1 [1.9;2.6] | 1.9 [1.9;2.3] | 2.3 [2.1;3.7] | 2.3 [1.9;2.9] | 2.2 [1.8;2.7] | 2.3 [1.9;3.7] |
| Trunk_ML_ | 1.0 [0.9;1.2] | 1.0 [0.9;1.6] | 1.0 [0.8;1.2] | 1.1 [0.8;1.4] | 0.9 [0.6;1.3] | 1.1 [1.1;1.4]^@^ | 1.1 [1.0;1.3] | 1.1 [0.8;1.3] | 1.1 [1.0;1.3] |
| Pelvis_AP_ | 1.7 [1.4;2.1] | 1.9 [1.6;2.5]^#^ | 1.4 [1.2;1.9] | 1.5 [1.4;2.0] | 1.4 [1.4;1.4]^&^ | 1.6 [1.5;2.3] | 1.6 [1.4;2.4] | 1.8 [1.4;2.7] | 1.6 [1.4;2.2]^&^ |
| Pelvis_ML_ | 0.7 [0.6;0.8] | 0.8 [0.7;0.9]* | 0.6 [0.5;0.7] | 0.6 [0.4;0.7] | 0.5 [0.4;0.6]^@^ | 0.7 [0.5;0.8] | 0.7 [0.4;0.9] | 0.7 [0.4;1.0] | 0.8 [0.5;0.8] |
| Hip_AP_ | 0.9 [0.8;1.4] | 0.9 [0.8;1.6] | 0.8 [0.5;1.3] | 1.0 [0.6;1.5] | 0.7 [0.6;1.0]^&^ | 1.3 [1.0;2.2]^@^ | 0.9 [0.7;1.3] | 0.9 [0.6;1.2] | 1.1 [0.7;1.8]^@^ |
| Hip_ML_ | 0.4 [0.4;0.6] | 0.5 [0.4;0.7] | 0.4 [0.3;0.6] | 0.4 [0.3;0.7] | 0.4 [0.3;0.4] | 0.5 [0.5;0.8] | 0.5 [0.3;0.6] | 0.4 [0.3;0.6] | 0.5 [0.4;0.6] |
| Knee_AP_ | 1.2 [0.9;2.2] | 1.3 [0.9;2.3] | 1.0 [0.6;2.0] | 1.3 [0.9;1.8] | 1.0 [0.8;1.3]^@^ | 1.8 [1.0;2.9] | 1.5 [1.0;1.8] | 1.5 [1.0;1.9] | 1.5 [0.8;1.9] |
| Knee_ML_ | 0.3 [0.2;0.6] | 0.3 [0.3;0.4] | 0.2 [0.2;0.7] | 0.3 [0.2;0.7] | 0.3 [0.2;0.4] | 0.6 [0.2;0.7] | 0.4 [0.3;0.9] | 0.4 [0.3;0.9] | 0.4 [0.2;0.8] |
| Ankle_AP_ | 1.2 [0.8;1.4] | 1.2 [0.9;1.6] | 0.9 [0.6;1.3] | 0.9 [0.7;1.5] | 0.9 [0.6;1.1]^@^ | 1.1 [0.7;2.5] | 1.1 [0.8;1.5] | 1.2 [0.7;1.5] | 1.0 [0.9;1.7]^@^ |
| Ankle_ML_ | 1.2 [1.0;1.5] | 1.3 [1.1;1.8]^#^ | 1.1 [0.8;1.3] | 1.0 [0.8;1.6] | 0.9 [0.8;1.3]^&^ | 1.7 [1.0;1.9] | 1.2 [0.8;1.4] | 1.2 [0.8;1.4]^@^ | 1.1 [0.9;1.4] |

^*^p<0.05, #p=0.05 comparison between FD and FI groups; ^@^p<0.05, ^&^p≤0.07 comparison between tSCS and control.

**Table S5.** Intersegmental cross-correlation coefficients (CC) and corresponding lags (in seconds) in control, T11 and L1 tSCS for the FD and FI groups in standing position.

| **Pair** | **Control** | | | **T11** | | | **L1** | | |
| --- | --- | --- | --- | --- | --- | --- | --- | --- | --- |
|  | FD+FI | FD | FI | FD+FI | FD | FI | FD+FI | FD | FI |
| Head-Trunk_AP_ (CC) | 0.2  [-0.2;0.5] | 0.2  [-0.3;0.4] | 0.4  [-0.2;0.4] | 0.2  [-0.2;0.3] | -0.2  [-0.3;0.4] | 0.2  [0.1;0.3] | 0.2  [-0.1;0.4] | 0.3  [-0.1;0.4] | 0.2  [-0.1;0.2] |
| *Head-Trunk_AP_ (lag)* | *-0.08*  *[-0.9;0.0]* | *-0.9*  *[-1.0;0.0]* | *-0.03*  *[0.0;0.1]* | *-0.15*  *[-0.2;1.0]* | *-0.08*  *[-0.1;0.9]* | *-0.15*  *[-0.3;1.0]* | *-0.05*  *[-0.1;0.2]* | *-0.01*  *[-0.2;0.3]* | *-0.05*  *[-0.1;0.0]* |
| Head-Trunk_ML_  (CC) | 0.5  [0.3;0.5] | 0.5  [0.3;0.5] | 0.4  [0.3;0.5] | 0.6  [0.5;0.7]^@^ | 0.6  [0.5;0.7]^@^ | 0.6  [0.4;0.7] | 0.5  [0.4;0.8] | 0.4  [0.4;0.7] | 0.6  [0.2;0.8] |
| *Head-Trunk_ML_*  *(lag)* | *0.0*  *[0.0;0.0]* | *0.0*  *[0.0;0.0]* | *0.0*  *[0.0;0.0]* | *0.03*  *[0.0;0.0]* | *0.02*  *[0.0;0.0]* | *0.05*  *[0.0;0.1]* | *0.03*  *[0.0;0.0]* | *0.03*  *[0.0;0.0]* | *0.02*  *[0.0;0.5]* |
| Trunk-RHip_AP_  (СС) | 0.2  [-0.2;0.2] | -0.1  [-0.4;0.2] | 0.2  [0.1;0.3] | 0.2  [0.1;0.3] | 0.1  [-0.1;0.3]^#^ | 0.3  [0.2;0.3] | 0.1  [-0.1;0.3] | 0.2  [0.0;0.3]^#^ | 0.1  [-0.1;0.1] |
| *Trunk-RHip_AP_*  *(lag)* | *0.1*  *[-0.1;0.9]* | *0.03*  *[-0.4;0.6]* | *0.3*  *[0.0;1.0]* | *-0.08*  *[-0.8;0.1]* | *-0.4*  *[-0.9;0]* | *0.0*  *[-0.1;0.2]* | *0.1*  *[-0.5;0.8]* | *0.1*  *[-0.1;0.8]* | *-0.1*  *[-0.5;0.7]* |
| Trunk-RHip_ML_ (СС) | -0.1  [-0.3;0.1] | 0.1  [-0.2;0.2] | -0.2  [-0.3;-0.1] | 0.1  [-0.2;0.1] | -0.1  [-0.2;0] | -0.1  [-0.3;0.1] | -0.2  [-0.3;0.1] | -0.1  [-0.2;0.1] | -0.2  [-0.3;0.1] |
| *Trunk-RHip_ML_ (lag)* | *0.1*  *[-0.1;0.2]* | *0.1*  *[0.0;0.5]* | *0.1*  *[-0.2;0.1]* | *-0.09*  *[-0.6;0.1]* | *-0.2*  *[-0.7;0.1]* | *-0.01*  *[-0.6;0.2]* | *0.01*  *[-0.5;0.3]* | *-0.06*  *[-0.4;0.3]* | *-0.1*  *[-0.4;0.2]* |
| Trunk-LHip_AP_ (CC) | 0.1  [-0.2;0.2] | 0.1  [-0.3;0.2] | 0.2  [0.1;0.2] | 0.2  [-0.1;0.2] | 0.2  [-0.1;0.3] | 0.2  [-0.1;0.2] | 0.2  [0.0;0.4] | 0.3  [0.2;0.5]^@^ | 0.1  [-0.1;0.2] |
| *Trunk-LHip_AP_ (lag)* | *0.1*  *[-0.6;0.2]* | *0.08*  *[-0.3;0.5]* | *0.1*  *[-1;0.2]* | *0.01*  *[-0.6;0.5]* | *0.01*  *[-0.1;0.2]* | *0.1*  *[-0.7;0.9]* | *0.01*  *[-0.3;0.8]* | *0.0*  *[-0.1;0.5]* | *0.4*  *[-0.7;0.8]* |
| Trunk-LHip_ML_ (CC) | 0.1  [-0.2;0.3] | 0.1  [-0.2;0.2] | 0.2  [0.1;0.3] | 0.2  [-0.1;0.3] | 0.1  [-0.1;0.2] | 0.3  [0.1;0.4] | 0.1  [-0.2;0.3] | 0.1  [-0.2;0.2] | 0.3  [-0.1;0.3] |
| *Trunk-LHip_ML_ (lag)* | *0.1*  *[-0.2;0.3]* | *0.01*  *[-0.3;0.3]* | *0.1*  *[0.01;0.2]* | *0.0*  *[-0.1;0.1]* | *-0.1*  *[-0.2;0.3]* | *0.02*  *[0;0.1]* | *-0.02*  *[-0.6;0.3]* | *0.0*  *[-0.4;0.4]* | *-0.04*  *[-0.7;0.1]* |
| RHip-RAnkle_AP_ (СС) | -0.1  [-0.4;0.2] | -0.2  [-0.4;0.2] | -0.1  [-0.3;0.3] | -0.2  [-0.3;0.2] | -0.2  [-0.4;0.0] | 0.1  [-0.3;0.4] | -0.2  [-0.4;0.0] | -0.3  [-0.5;0.0] | -0.2  [-0.4;0.0] |
| *RHip-RAnkle_AP_ (lag)* | *-0.01*  *[-0.1;0.4]* | *0.0*  *[-0.1;0.5]* | *-0.01*  *[0.0;0.4]* | *-0.03*  *[-0.2;0.1]* | *-0.03*  *[-0.1;0.0]* | *-0.07*  *[-0.3;0.5]* | *0.09*  *[0.0;0.2]* | *-0.1*  *[0.0;0.4]* | *0.05*  *[0.0;0.1]* |
| RHip-RAnkle_ML_ (CC) | -0.4  [-0.5;-0.2] | -0.5  [-0.6;-0.3] | 0.2  [0.1;0.3]* | -0.3  [-0.5;0.1] | -0.4  [-0.5;-0.2] | 0.1  [-0.2;0.2] | -0.3  [-0.5;0.2] | -0.4  [-0.5;-0.1] | 0.1  [-0.5;0.2] |
| *RHip-RAnkle_ML_*  *(lag)* | *-0.06*  *[-0.2;0.1]* | *0.0*  *[-0.1;0.2]* | *-0.2*  *[-0.3;-0.1]* | *0.0*  *[-0.4;0.2]* | *0.0*  *[-0.2;0.1]* | *0.02*  *[-0.4;1.0]* | *-0.05*  *[-0.2;0.0]* | *-0.09*  *[-0.3;0.0]* | *-0.01*  *[-0.1;1.0]* |
| LHip-LAnkle_AP_ (CC) | -0.1  [-0.3;0.2] | -0.2  [-0.3;0.2] | 0.2  [-0.4;0.2] | -0.3  [-0.4;0.0] | -0.3  [-0.3;-0.1] | -0.3  [-0.4;0.1]^#^ | -0.2  [-0.3;0.2] | 0.1  [-0.2;0.2] | -0.3  [-0.4;-0.1]^#^ |
| *LHip-LAnkle_AP_ (lag)* | *0.1*  *[-0.3;1.0]* | *0.2*  *[-0.5;1.0]* | *0.1*  *[0.0;1.0]* | *0.0*  *[0.0;0.2]* | *0.0*  *[0.0;0.2]* | *0.0*  *[-0.1;0.1]* | *-0.05*  *[-1.0;0.3]* | *-0.1*  *[-1.0;0.4]* | *-0.02*  *[-0.1;0.3]* |
| LHip-LAnkle_ML_ (CC) | -0.4  [-0.7;-0.1] | -0.5  [-0.6;0.2] | -0.2  [-0.7;-0.1] | -0.4  [-0.5;-0.2] | -0.5  [-0.6;-0.3] | -0.3  [-0.4;-0.1] | -0.5  [-0.6;0.0] | -0.6  [-0.6;-0.2] | -0.3  [-0.5;0.2] |
| *LHip-LAnkle_ML_ (lag)* | *0.0*  *[0.0;0.1]* | *0.0*  *[0.0;0.1]* | *0.0*  *[0.0;0.1]* | *-0.1*  *[-0.8;0.0]* | *-0.07*  *[-0.3;0.0]* | *-0.5*  *[-0.9;-0.2]* | *0.0*  *[0.0;0.3]* | *0.0*  *[0.0;0.1]* | *0.0*  *[0.0;0.4]* |

*p<0.05 comparison between FD and FI groups, ^@^p<0.05, ^#^p≤0.06 comparison between tSCS and control.

**Table S6.** Cross-correlation coefficients (CC) and corresponding lags (in seconds) between hips and COP frontal motion and between ankles and COP sagittal motion in control, T11 and L1 tSCS for the FD and FI groups in standing position.

| **Pair** | **Control** | | | **T11** | | | **L1** | | |
| --- | --- | --- | --- | --- | --- | --- | --- | --- | --- |
|  | FD+FI | FD | FI | FD+FI | FD | FI | FD+FI | FD | FI |
| RHip-COP_ML_ (CC) | -0.5  [-0.6;-0.4] | -0.6  [-0.7;-0.4] | -0.4  [-0.5;-0.2]^#^ | -0.5  [-0.6;-0.3] | -0.6  [-0.7;-0.4] | -0.4  [-0.6;-0.3] | -0.5  [-0.7;-0.3] | -0.6  [-0.7;-0.4] | -0.3  [-0.7;0.1] |
| *RHip-COP_ML_* *(lag)* | *-0.18*  *[-0.2;0.0]* | *-0.18*  *[-0.2;0.0]* | *-0.17*  *[-0.2;0.0]* | *-0.14*  *[-0.2;0.0]* | *-0.12*  *[-0.2;0.0]* | *-0.14*  *[-0.2;0.0]* | *-0.01*  *[-0.1;0.0]* | *0.0*  *[0.0;0.1]* | *-0.10*  *[-0.2;0.0]* |
| LHip-COP_ML_ (CC) | 0.5  [0.3;0.7] | 0.6  [0.4;0.7] | 0.3  [0.3;0.5]^#^ | 0.5  [0.4;0.7] | 0.6  [0.4;0.7] | 0.4  [0.3;0.5] | 0.5  [0.3;0.7] | 0.6  [0.4;0.7] | 0.3  [0.2;0.6] |
| *LHip-COP_ML_* *(lag)* | *-0.07*  *[-0.2;0.1]* | *-0.06*  *[-0.2;0.1]* | *-0.09*  *[-0.2;0.1]* | *-0.10*  *[-0.2;0.0]* | *-0.09*  *[-0.2;0.0]* | *-0.13*  *[-0.2;-0.1]* | *-0.05*  *[-0.1;0.0]* | *0.0*  *[0.0;0.1]* | *-0.15*  *[-0.2;-0.1]* |
| RAnkle-COP_AP_ (CC) | -0.7  [-0.8;-0.5] | -0.7  [-0.8;-0.5] | -0.6  [-0.7;-0.6] | -0.6  [-0.8;-0.5] | -0.6  [-0.8;-0.5] | -0.7  [-0.7;-0.5] | -0.6  [-0.7;-0.5] | -0.6  [-0.8;-0.5] | -0.6  [-0.7;-0.4] |
| *RAnkle-COP_AP_* *(lag)* | *-0.01*  *[-0.1;0.1]* | *0.02*  *[0.0;0.1]* | *-0.03*  *[-0.1;0.1]* | *-0.03*  *[-0.1;0.1]* | *-0.11*  *[-0.1;0.0]* | *0.0*  *[0.0;0.1]* | *0.0*  *[0.0;0.1]* | *0.0*  *[-0.1;0.1]* | *0.0*  *[0.0;0.0]* |
| LAnkle-COP_AP_ (CC) | -0.7  [-0.8;-0.6] | -0.7  [-0.8;-0.6] | -0.7  [-0.8;-0.7] | -0.7  [-0.8;-0.6] | -0.8  [-0.8;-0.6] | -0.7  [-0.8;-0.6] | -0.7  [-0.8;-0.7] | -0.8  [-0.8;-0.6] | -0.7  [-0.8;-0.7] |
| *LAnkle-COP_AP_* *(lag)* | *0.0*  *[-0.1;0.0]* | *0.0*  *[-0.1;0.0]* | *0.0*  *[-0.1;0.0]* | *0.0*  *[0.0;0.1]* | *-0.01*  *[-0.1;0.0]* | *0.01*  *[0.0;0.1]* | *0.0*  *[-0.1;0.0]* | *0.0*  *[-0.1;0.0]* | *0.0*  *[0.0;0.0]* |

^#^p=0.06 comparison between FD and FI groups.

**Table S7.** Respiratory parameters in control and tSCS conditions for FD and FI groups in sitting and standing positions.

| **Parameter** | **Control** | | | **T11** | | | **L1** | | |
| --- | --- | --- | --- | --- | --- | --- | --- | --- | --- |
|  | FD+FI | FD | FI | FD+FI | FD | FI | FD+FI | FD | FI |
|  | Standing | | | | | | | | |
| BR, bpm | 17 [16;19] | 17 [16;19] | 17 [14;18] | 17  [14;20] | 19  [14;20] | 17  [15;18] | 17  [15;20] | 19  [15;20] | 16  [15;17] |
| T_in_, sec | 1.3 [1.2;1.5] | 1.3 [1.2;1.4] | 1.4 [1.2;1.6] | 1.3 [1.2;1.6] | 1.2 [1.1;1.6] | 1.6 [1.2;1.6] | 1.3 [1.2;1.5] | 1.3 [1.1;1.4] | 1.5 [1.2;1.5] |
| T_ex_, sec | 2.1 [1.8;2.2] | 2.1 [1.6;2.1] | 2.1 [1.9;2.4] | 2.0 [1.7;2.4] | 1.9 [1.7;2.4] | 2.2 [1.9;2.7] | 2.1 [1.7;2.3] | 1.8 [1.7;2.3] | 2.1 [2.0;2.3] |
|  | sitting | | | | | | | | |
| BR, bpm | 18 [17;19] | 18 [17;19] | 17 [16;19] | 17  [16;19] | 17  [16;19] | 18  [17;20] | 18  [17;19] | 18  [17;18] | 18  [17;19] |
| T_in_, sec | 1.3 [1.2;1.4] | 1.3 [1.2;1.4] | 1.2 [1.2;1.5] | 1.3 [1.2;1.4] | 1.3 [1.2;1.4] | 1.3 [1.1;1.3] | 1.3 [1.2;1.4] | 1.3 [1.2;1.4] | 1.2 [1.2;1.4] |
| T_ex_, sec | 1.9 [1.8;2.0] | 2.0 [1.8;2.0] | 1.9 [1.8;2.2] | 2.0 [1.8;2.1] | 2.1 [1.9;2.2] | 2.0 [1.7;2.1] | 1.9 [1.8;2.0] | 1.9 [1.8;2.0] | 1.9 [1.7;1.9] |


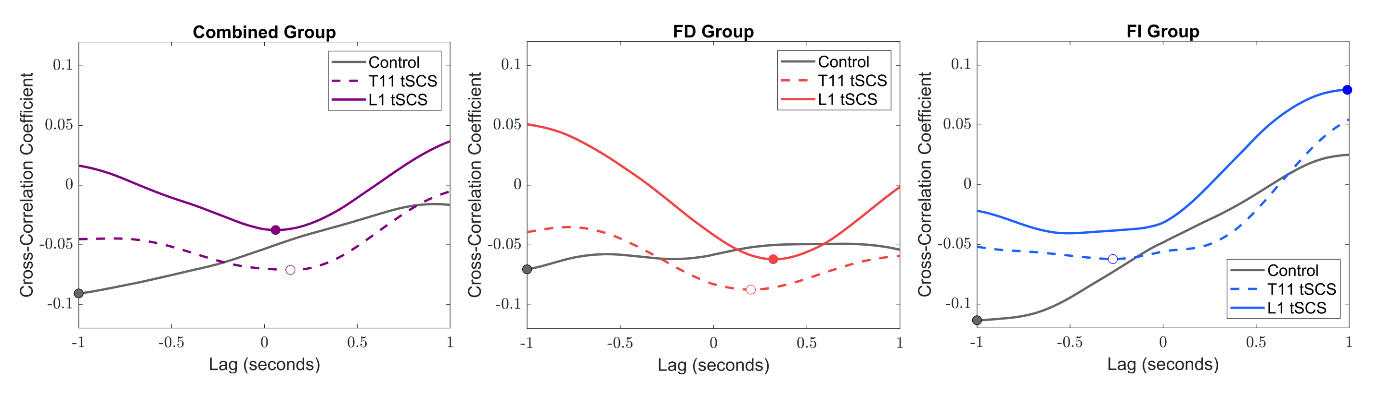
 **Figure S1.** Averaged cross-correlation functions and corresponding lags between respiratory curve and CoP displacement in the AP direction in the combined group, in the FD group and in the FI group in control, T11 and L1 tSCS standing.
